# Automated Sequential Analysis of Hydrophilic and Lipophilic Fractions of Biological Samples: Increasing Single-Injection Chemical Coverage in Untargeted Metabolomics

**DOI:** 10.3390/metabo11050295

**Published:** 2021-05-05

**Authors:** Kristian Pirttilä, Göran Laurell, Curt Pettersson, Mikael Hedeland

**Affiliations:** 1 Department of Medicinal Chemistry, Uppsala University, SE-75123 Uppsala, Sweden; curt.pettersson@ilk.uu.se (C.P.); mikael.hedeland@ilk.uu.se (M.H.); 2 Department of Surgical Science, Uppsala University, SE-75185 Uppsala, Sweden; goran.laurell@surgsci.uu.se

**Keywords:** LC–MS, sequential columns, automated, chemical coverage, untargeted metabolomics

## Abstract

In order to increase metabolite coverage in LC–MS-based untargeted metabolomics, HILIC- and RPLC-mode separations are often combined. Unfortunately, these two techniques pose opposite requirements on sample composition, necessitating either dual sample preparations, increasing needed sample volume, or manipulation of the samples after the first analysis, potentially leading to loss of analytes. When sample material is precious, the number of analyses that can be performed is limited. To that end, an automated single-injection LC–MS method for sequential analysis of both the hydrophilic and lipophilic fractions of biological samples is described. Early eluting compounds in a HILIC separation are collected on a trap column and subsequently analyzed in the RPLC mode. The instrument configuration, composed of commercially available components, allows easy modulation of the dilution ratio of the collected effluent, with sufficient dilution to obtain peak compression in the RPLC column. Furthermore, the method is validated and shown to be fit for purpose for application in untargeted metabolomics. Repeatability in both retention times and peak areas was excellent across over 140 injections of protein-precipitated blood plasma. Finally, the method has been applied to the analysis of real perilymph samples collected in a guinea pig model. The QC sample injections clustered tightly in the PCA scores plot and showed a high repeatability in both retention times and peak areas for selected compounds.

## 1. Introduction

One of the main issues that still plague untargeted metabolomics is the immense chemical diversity as well as the wide concentration range of the metabolites that make up biological samples [1,2]. Achieving complete chemical coverage with a single analytical technique is a practical impossibility as each technique comes with its own inherent limitations. With this in mind, the aim in developing an analytical method for untargeted metabolomics instead becomes that of maximizing the chemical coverage obtained within the bounds of the techniques employed.

One approach to increase the chemical coverage obtained in an untargeted metabolomics study is to combine different analytical techniques that provide complementary information [3,4,5]. One of the most powerful of such techniques used today is liquid chromatography hyphenated with high-resolution mass spectrometry (LC–HRMS) [6,7]. This is due to the large variation of possible combinations of columns and mobile phases, giving the separation technique the potential to cover a very wide range of chemical properties. A common combination of separation modes is HILIC and RPLC, as they are, to a large extent, complementary in terms of their chemical polarities [6,8]. However, an immediate consequence of combining these separation modes is the need to either (i) split or (ii) evaporate and re-dissolve the samples as the two techniques pose opposite requirements on the injection solvent used. In order to split the samples, a relatively large sample volume is needed and further processing may result in loss of analytes. In addition, the combination of different techniques also increases the total number of analyses performed per sample, leading to a higher demand on instrument time, thereby increasing costs.

The issue of limited sample volume was highlighted to us in a recent study, where we investigated the attenuating effect of hydrogen gas on noise-induced hearing loss (NIHL) in guinea pigs. The sample material used in the study was perilymph, sampled from the inner ear [9,10,11]. In the case of perilymph, the maximum volume that can safely be aspirated, without contamination from cerebrospinal fluid (CSF), is less than 1 µL [12]. For this reason, the samples in the above-mentioned study were analyzed using only HILIC chromatography due to its high compatibility with small water-soluble compounds [6]. In order to increase the chemical coverage obtained in such studies, novel analytical methods are needed.

One approach to this problem is to utilize so-called column-switching techniques. These have been employed for several applications in the past, including online SPE [13,14], serial column coupling [15,16,17,18], parallel column coupling [19], and comprehensive 2DLC [20]. Such methods can also been designed for sequential column coupling, where the poorly retained compounds on a primary column are collected and subsequently separated using a secondary column (e.g., HILIC followed by RPLC). The main issue with this approach, when using HILIC and RPLC, is that the early effluent in a HILIC separation would need to be diluted extensively to reduce the acetonitrile content and thereby the elution strength in the RPLC mode. A number of approaches to this problem have been previously presented but very few have been focused on metabolomics. However, in 2008, Wang et al. presented a method utilizing a large volume mixer preloaded with diluent [21] where the aim was to increase chemical coverage in metabolomics. The collected effluent from a HILIC separation was introduced into the mixer allowing the relative acetonitrile content to be reduced. Similarly, Kittlaus et al. published a paper in 2013 that used two binary pumps where a primary pump was used to elute the first column and the secondary pump was used to dilute the effluent and elute the secondary column for residue analysis of pesticides [22]. Another example of a similar solution was published in 2014 by Cabooter et al. that also utilized static mixers for dilution of the collected fraction [23]. In this study, the focus was to develop methods for the analysis of pharmaceuticals in environmental samples. The protocol of Cabooter et al. was later refined by Loos et al. in 2017 who demonstrated a novel mixing unit utilizing what they called restriction capillaries [24]. A somewhat different approach was taken by Wang et al. in 2017 who used a pre-column to separate the lipidome from the metabolome for sequential lipidomics/metabolomics analysis [25]. To facilitate a time-efficient and practical solvent switching, the methods mentioned above predominantly use a narrower diameter in the primary than the secondary column. Such an approach sacrifices the benefits of using a 2.1 mm i.d. UHPLC column in the secondary separation, e.g., increased sensitivity due to reduced radial dilution and increased chromatographic efficiency, as well as less solvent waste. Thus, there is a need for further development of separation methods with a high chemical coverage aimed at untargeted metabolomics.

The aim of this work was to develop a method with increased chemical coverage per injection for use in untargeted metabolomics studies where available sample volume is limited. To that end, herein is presented an LC–MS-based procedure that allows the poorly retained compounds eluting close to the solvent front in a HILIC separation to be collected and analyzed in the RPLC mode after the HILIC separation is concluded. This allows for sequential analysis of compounds with a wide range of chemical polarities using a single injection. The instrumental setup is composed of commercially available components that can be easily installed and configured. In addition, the instrument is setup to allow easy modulation of the dilution ratio. The developed method is shown to be fit for purpose for use in untargeted metabolomics and applied to the analysis of guinea pig perilymph in a follow-up study of hydrogen gas on NIHL.

## 2. Results and Discussion

As previously stated, the trapping of compounds in early-gradient HILIC effluent on a reversed-phase trap column is challenging due to the high acetonitrile content. For this reason, the focus was finding an instrumental configuration that would allow sufficient dilution of the collected volume while maintaining most of the separation efficiency of a conventional single-column separation.

### 2.1. Instrumental Configuration

**Figure 1:**
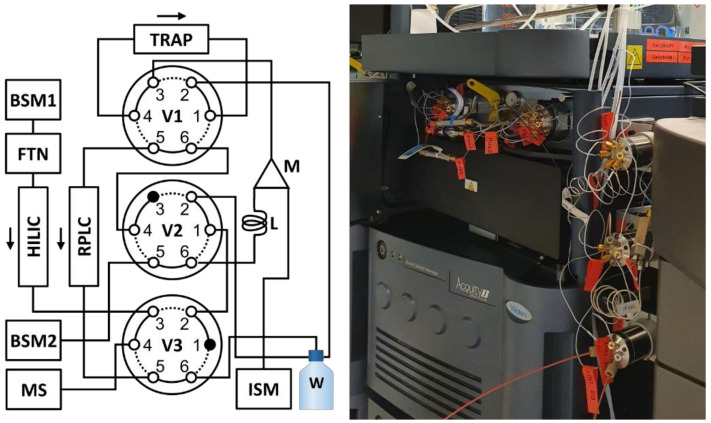
Instrumental configuration for the sequential analysis of both hydrophilic and lipophilic compounds in biological samples (**left**) and a picture of the valves and adapted column manager (**right**). The instrument is composed of two binary pumps (BSM1 and BSM2), an isocratic pump (ISM), a column manager configured to allow selection of various trap columns (TRAP), a flow-through-needle sample manager (FTN), a 200 µL sample loop (L), a 50 µL static mixing unit (M), three 6-port VICI valves (V1-3), and a Synapt G2S HRMS instrument (MS). The column manager ovens were used to control the temperature of the analytical columns.

A scheme of the final configuration is presented in Figure 1. Using three 6-port VICI valves (V1-3, Figure 1) in combination with two binary pumps (BSM1 and BSM2, Figure 1) and one isocratic pump (ISM, Figure 1), the instrument could be configured to use the BSM2 pump to achieve the necessary dilution of the collected effluent. All components used are commercially available. In short, the HILIC effluent is redirected to a 200 µL sample loop (L, Figure 1) at a pre-specified time point and collected. After collection, the content of the sample loop is slowly displaced into the mixer (M, Figure 1) using a low flow of 100% aqueous phase from BSM2. Here, it is combined with a high flow of 100% aqueous phase delivered by the ISM pump. This allows for a tunable dilution ratio by modulating the relative flow rates of BSM2 and ISM. As an example, a 0.020 mL/min flow from the BSM2 pump and a 1.9 mL/min flow from the ISM pump will result in a dilution of 95 times. See section Materials and Methods for a more detailed description of the method.

### 2.2. Choice of Heart-Cut Dilution Method

As mentioned earlier, a number of methods for the dilution of the collected fraction have been presented in the past. The use of large volume mixers [21,23] is a straight forward approach. However, it leads to very bulky and expensive configurations and was not tested in this work. The method presented by Loos et al. in 2017 [24], using restriction capillaries to obtain a flow differential, showed promise as a possible alternative and was initially compared to the direct approach using the BSM2 pump presented in this work (Figure 1). As far as technical aspects go, both methods performed well and without any direct issues. However, the initially selected dilution ratio (approximately 74 times, as suggested in the paper by Loos et al.) was not sufficient to trap the moderately polar compounds in our standard set (data not shown). Changing the dilution ratio when using restriction capillaries requires replacing one or both of the capillaries. This led us to focus on the more flexible approach presented in the previous section as this allowed easier adjustment of the dilution ratio.

### 2.3. Conditions Related to Trapping of the Collected Compounds

#### 2.3.1. Selection of Trap Column Stationary Phase

A number of trap columns have been evaluated in terms of their ability to retain the test compounds used in developing this method. This has been assessed in terms of the peak efficiency of the trapped compounds as well as by relative peak area against a direct injection to the RPLC column. The tested trap columns were an Xbridge C8, an Xbridge C18, an HSS T3, an Xbridge phenyl, and an Oasis HLB. As far as retaining the more lipophilic compounds, such as diclofenac, any of the tested trap columns performed well (data not shown). The issue has mainly been to retain the compounds with chemical polarity in the borderland between the compatibility ranges of RPLC and HILIC. This requires that the trap column is general enough to retain both these borderland compounds as well as more lipophilic compounds. The only trap column, of the ones tested, that showed such generality was the Oasis HLB column, and was therefore our choice for the final method.

#### 2.3.2. Investigation of Necessary Dilution Factor

**Figure 2:**
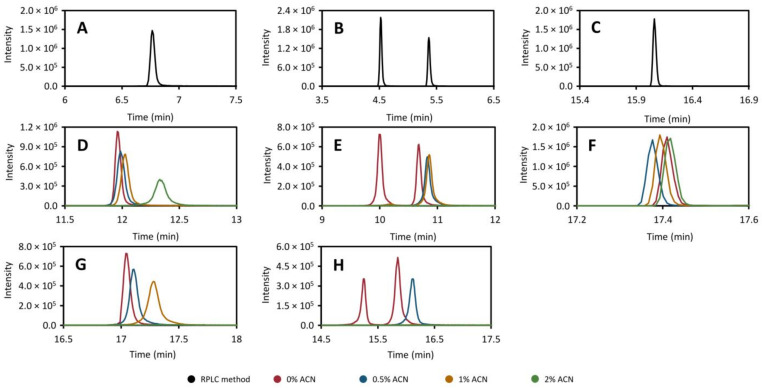
The effect of acetonitrile content on the peak efficiency and recovery of caffeine (*m/z* 195.088, (**A**,**D**,**G**)), theobromine (left peak) and theophylline (right peak) (*m/z* 181.072, (**B**,**E**,**H**)) and diclofenac (*m/z* 214.042, (**C**,**F**)) trapped in the heart-cut method in contrast with a direct injection onto the RPLC column with a conventional separation (**A**–**C**), black. In this experiment, the HILIC column was removed and the standard mixture was injected directly towards the trap column into a flow with 0% (red), 0.5% (blue), 1% (yellow), or 2% (green) acetonitrile. The flow was then kept running for five (**D**–**F**) or ten minutes (**G**–**H**) after which the trap column was back-flushed to the RPLC column using gradient elution as described in Section 3.5. In the 10 min experiment, the retention time of diclofenac was longer than the method.

As mentioned above, there are compounds that fall on the border between HILIC and RPLC, in terms of retention. In order to better understand the magnitude of the effect that residual acetonitrile has on the trapping phase of the method, we removed the HILIC column and performed a number of injections of the standard mix (dissolved in 95:5 acetonitrile: water, *v/v*) directly into a continuous flow of solvent with varying acetonitrile content going through the trap column (Figure 2, red line: 0%, blue line: 0.5%, orange line: 1%, and green line: 2%). In addition, to get an idea of the effect of the total loading time, the flow was allowed to continue through the trap column for five (Figure 2D–F) or ten minutes (Figure 2G,H) after the injection. The results of these experiments are presented in Figure 2, which show the chromatograms of caffeine (Figure 2A,D,G), theophylline (Figure 2B,E,H), theobromine (Figure 2B,E,H), and diclofenac (Figure 2C,F) injected directly onto the trap column (Figure 2D–H) as well as a normal direct injection onto the RPLC column (Figure 2A–C). It is clear that the acetonitrile content has a major impact on both the total recovery and peak shape of the moderately polar compounds (e.g., theophylline, theobromine, and caffeine, Figure 2). For the more lipophilic compounds (see diclofenac in Figure 2), increased acetonitrile content did not have a detrimental effect on the peak shape when pumping for 5 min. Unfortunately, when pumping was continued for 10 min, the retention time of diclofenac was longer than the data collection method, thereby no data are available in this case. Worth noting here is that, with acetonitrile levels at or above 1%, the theophylline peak disappears completely even at loading times of 5 min. The effect on peak shapes is pronounced even at 0.5% acetonitrile, regardless of loading time. One conclusion that can be drawn from this is that the collected fraction has to be diluted as much as possible within the instrumental constraints (preferably to <1% acetonitrile content) if the moderately polar compounds are to be trapped successfully.

### 2.4. Selection of HILIC Separation Conditions

The HILIC method used was adapted from an earlier protocol used for untargeted metabolomics in our lab [9,10]. Adjustments to the method were primarily aimed at minimizing the retention of the compounds that would be collected in the heart-cut in order to minimize the collected volume. To that end, the mobile-phase gradient was started at a slightly lower acetonitrile content (95% A) than in the original method (100% A).

**Figure 3:**
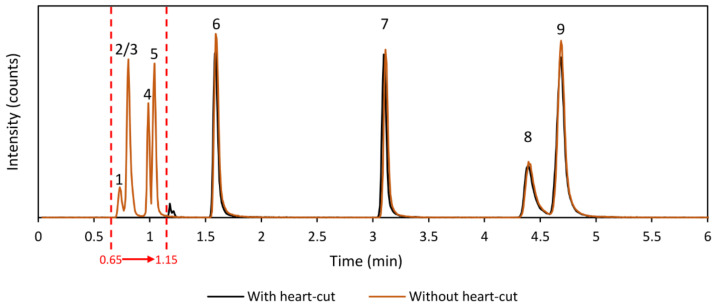
Summed extracted ion chromatograms of diclofenac (1, *m/z* 214.042), caffeine (2, *m/z* 195.088), 2-aminobenzoic acid (3, *m/z* 120.045), theophylline (4, *m/z* 181.072), theobromine (5, *m/z* 181.072), acetylcholine (6, *m/z* 146.117), serotonin (7, *m/z* 160.076), phenylalanine (8, *m/z* 120.081), and acetylcarnitine (9, *m/z* 204.123) showing the effect of valve switching on the HILIC separation with- (black trace) and without (orange trace) a heart-cut being made. In the heart-cut experiment (black trace), the valve is switched between 0.65 min and 1.15 min to direct the HILIC effluent to the sample loop (indicated with red dashed lines). When the heart-cut is performed, peaks 1–5 are missing as these compounds as diverted away from the detector (black trace). No large effects are seen in the peak shapes, retention time, or response of the compounds analyzed using HILIC in the heart-cut method (compounds 6–9).

It is important to ensure that the HILIC separation is not negatively affected by the switching of the valves. Therefore, the HILIC separation has been monitored in every step of developing the method to ensure that it remains unaffected in terms of separation performance and peak shapes. A comparative example chromatogram of the HILIC separation with and without performing the heart-cut is shown in Figure 3 and we conclude that the valve-switching events do not impair the HILIC chromatography to a measurable degree.

### 2.5. Optimization of RPLC Parameters with Backflush of the Trap Column

Experiments have shown that the trapped compounds elute off the trap column in broad bands. Thus, the focusing of the trapped compounds appears to occur mostly at the head of the secondary column, rather than on the trap column (see Supplementary Materials Figure S1). As such, it can be concluded that the initial conditions in the elution of the trap column and RPLC separation plays a key role in the final outcome of the analysis and should therefore be carefully optimized. To that end, the flow rate, column temperature, and gradient were optimized for the secondary separation on the RPLC column.

#### 2.5.1. Flow Rate in the RPLC Separation

**Figure 4:**
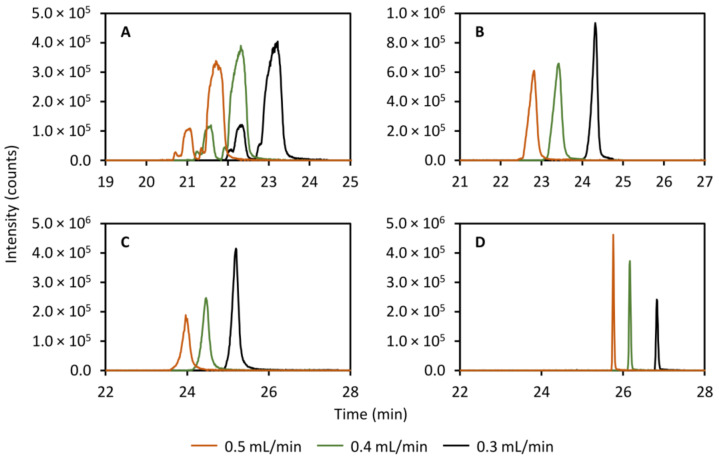
Effect of varying the flow rate in the secondary column separation on the peak shapes of theobromine (*m/z* 181.072, left peak, (**A**)), theophylline (*m/z* 181.072, right peak, **A**), caffeine (*m/z* 195.088, (**B**)), 2-aminobenzoic acid (*m/z* 120.045, (**C**)), and diclofenac (*m/z* 214.042, (**D**)). A clear effect is observed for caffeine and 2-aminobenzoic acid, yielding narrower peaks with a decreased flow rate.

The flow rate in the secondary column was tested between 0.3 mL/min and 0.5 mL/min and the results are presented in Figure 4 for theobromine (left peak, panel A, Figure 4), theophylline (right peak, panel A, Figure 4), caffeine (panel B, Figure 4), 2-aminobenzoic acid (panel C, Figure 4), and diclofenac (panel D, Figure 4). Here, it is clear that a flow rate of 0.3 mL/min produces the sharpest peaks for caffeine and 2-aminobenzoic acid. The peaks of theobromine and theophylline exhibit very poor efficiency and there is no clear effect of changing the flow rate. As far as diclofenac goes, its peak instead appears to get slightly broader with lower flow rates but the effect is very small.

#### 2.5.2. Temperature of the RPLC Column

**Figure 5:**
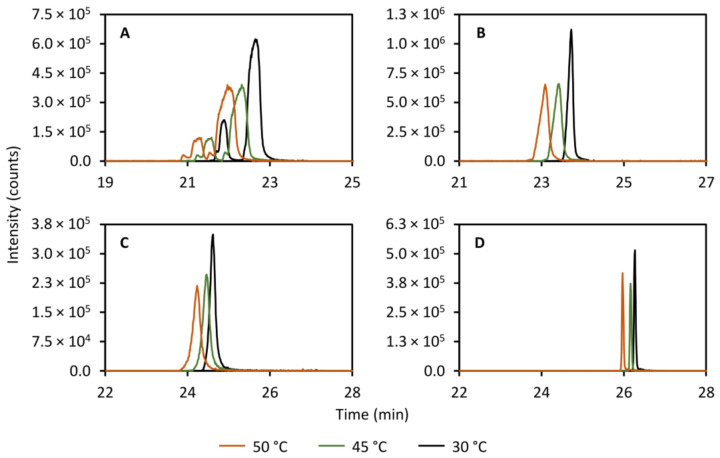
Effect of varying the column temperature in the secondary column separation on the peak shapes of theobromine (*m/z* 181.072, left peak, (**A**)), theophylline (*m/z* 181.072, right peak, (**A**)), caffeine (*m/z* 195.088, (**B**)), 2-aminobenzoic acid (*m/z* 120.045, (**C**)), and diclofenac (*m/z* 214.042, (**D**)). A column temperature of 30.0 °C yields the narrowest peaks for all compounds studied.

Another important parameter to study is the column temperature as this has an effect on multiple thermodynamic parameters such as equilibrium constants and the solvent viscosity, and thereby affects the partitioning of the solutes between the stationary phase and the mobile phase. Figure 5 shows the effect of varying the column temperature of the secondary column on the peak shapes of theobromine (left peak, panel A, Figure 5), theophylline (right peak, panel A, Figure 5), caffeine (panel B, Figure 5), 2-aminobenzoic acid (panel C, Figure 5), and diclofenac (panel D, Figure 5). As expected, a clear decrease in peak width is observed when lowering the temperature from 50.0 to 30.0 °C. As such, in order to maximize performance, the column temperature of the reversed-phase column was controlled at 25.0 °C, a few degrees above ambient room temperature.

#### 2.5.3. Gradient in the RPLC Separation

**Figure 6:**
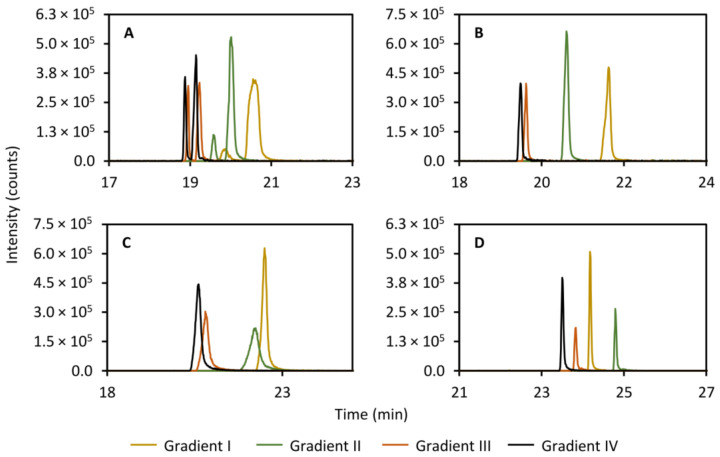
Effect of varying the gradient steepness in the secondary column separation on the peak shape of theobromine (*m/z* 181.072, left peak, (**A**)), theophylline (*m/z* 181.072, right peak, (**A**)), caffeine (*m/z* 195.088, (**B**)), 2-aminobenzoic acid (*m/z* 120.045, (**C**)), and diclofenac (*m/z* 214.042, (**D**)). The initial steepness of the gradients increase from gradient I to IV. The mobile phases were (A) 95:5 acetonitrile:water (*v/v*) with 5 mM ammonium formate and 0.065 vol% formic acid and (B) 5:95 acetonitrile:water (*v/v*) with 5 mM ammonium formate and 0.065 vol% formic acid. Gradient I: 0.00–1.00 min 100%A (isocratic), 1.00–5.00 min 100%A–85%A (linear gradient), 5.00–9.00 min 85%A–2%A (linear gradient). Gradient II: 0.00–1.00 min 100%A (isocratic), 1.00–5.00 min 100%A–70%A (linear gradient), 5.00–9.00 min 70%A–2%A (linear gradient). Gradient III: 0.00–1.00 min 100%A (isocratic), 1.00–5.00 min 100%A–50%A (linear gradient), 5.00–9.00 min 50%A–2%A (linear gradient). Gradient IV: 0.00–1.00 min 100%A (isocratic), 1.00–9.00 min 100%A–2%A (linear gradient).

Since the focusing appears to occur on the RPLC column rather than in the trap column, the gradient used in the analysis of the trapped compounds was expected to have a large impact on peak efficiency. Thus, it stands to reason that the time it takes to elute the compounds off the trap column should be minimized. The initial conditions were screened and it was shown that a preliminary isocratic period with 100% aqueous phase produced a marginal improvement to peak performance (results not shown) in contrast to starting the gradient directly. However, the overall largest improvement in peak performance of all screened parameters was observed when the gradient steepness was increased in the secondary column separation. Figure 6 shows the effect of increasing the initial steepness of the gradient on the peak efficiency of theobromine (left peak, panel A, Figure 6), theophylline (right peak, panel A, Figure 6), caffeine (panel B, Figure 6), 2-aminobenzoic acid (panel C, Figure 6), and diclofenac (panel D, Figure 6). A probable explanation to this is that the steeper gradient allows for more focusing to occur in the trap column before the compounds have time to elute to the RPLC column. However, this has not been confirmed experimentally.

### 2.6. Validation of the Method for Untargeted Metabolomics

The term validation is here used tentatively as no full validation according to regular analytical chemistry practice has been performed. We are instead striving to show that the method is fit for purpose to be used in untargeted metabolomics with only relative quantification where all the samples are analyzed sequentially. To that end, the parameters repeatability, carry over, and linearity were deemed of high importance in addition to instrumental metrics such as back pressure stability. For quantitative work with similar methods, we would strongly recommend a full validation. As the complexity of the chromatographic system increases, so does the risk for failure and drift in performance. As such, the focus of our validation was to ensure that the system would remain stable throughout a long analytical run, despite the added complexity from the additional components in the LC–MS system in contrast with a conventional system. Unfortunately, validating an untargeted method is a difficult task as it is impossible to ensure that all compounds detectable in the sample are performing to specification. In lieu of untargeted validation methods, a commonly used strategy [3,9,26,27] is to monitor selected compounds known to be present in the samples. These then serve as proxy measurements of performance for all compounds present. To that end, the repeatability was assessed for a number of known compounds, spiked into the samples, in terms of drift and variation in retention times and absolute peak areas. In addition, parameters related to instrumental performance was also monitored, such as back pressure and carry over. The developed method is ultimately intended to be used to analyze a set of guinea pig perilymph samples collected in a follow-up study of the attenuating effect of hydrogen gas on noise-induced hearing loss in guinea pigs.

#### 2.6.1. Choice of Sample Material

As perilymph is a highly precious sample material, finding the necessary volumes for a full validation run was not possible and as such, blood plasma was used instead. These fluids are generally considered to be similar in composition [28,29,30,31]. However, perilymph has lower concentrations, especially in terms of protein content. Thus, it is our belief that if the system can manage protein-precipitated blood plasma, it would also be able to handle protein-precipitated perilymph.

#### 2.6.2. Choice of Standard Compounds

The selection of suitable compounds to be used as standards in projects such as this is a difficult task as those that will be collected in the heart-cut have to have close to no retention in the HILIC mode (which is surprisingly difficult to find among small metabolites) and be sufficiently retained on an RPLC column. A total of 91 compounds from various chemical classes were screened in this work (see Supplementary Materials Table S4 for a full list of compounds screened). After removing those that were not deemed suitable due to too high retention in HILIC, too low retention in RPLC, not sufficient ionization, etc. the selection of suitable compounds were found to be caffeine, theobromine, theophylline, diclofenac, and 2-aminobenzoic acid. These compounds represent both the borderline compounds that are poorly retained in our HILIC method as well as exhibiting only moderate retention in the RPLC method along with those that are more clear-cut cases of no retention in the HILIC mode but high retention in the RPLC mode. To monitor the performance of the HILIC separation acetylcholine, acetylcarnitine, serotonin, and phenylalanine was used.

#### 2.6.3. Conditioning of the System

**Figure 7:**
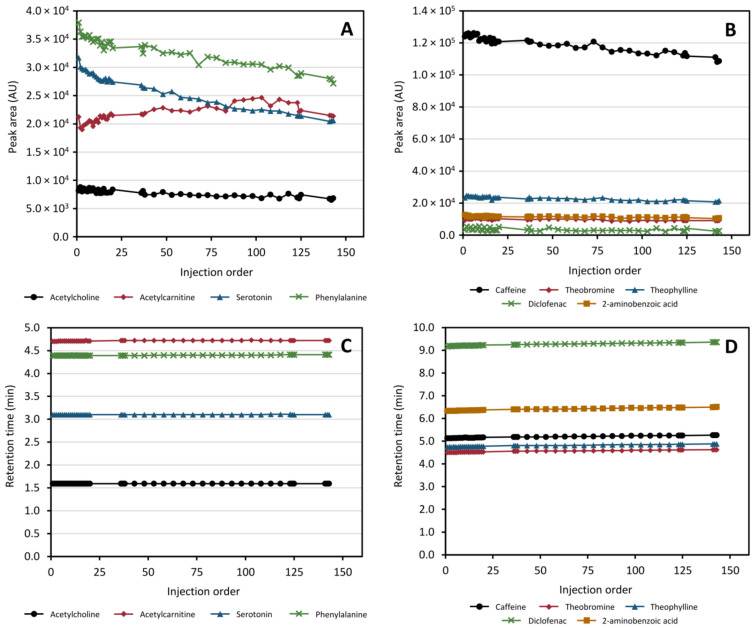
Peak area and retention time stability of acetylcholine, acetylcarnitine, serotonin, phenylalanine, caffeine, theobromine, theophylline, diclofenac, and 2-aminobenzoic acid in the heart-cut method throughout a simulated analytical run with spiked protein-precipitated plasma. Panels (**A**,**B**) display the peak area evolution over the run for the HILIC- and RPLC separation, respectively, while panels (**C**,**D**) display the retention time evolution for the HILIC- and RPLC separation, respectively.

It has been shown multiple times that it is necessary to “condition” the chromatographic system prior to running an analytical batch. It has been speculated that this achieves a coating of active sites in the column as well as equilibration of the system as a whole [26,27]. To investigate how many injections were needed, 20 repeated injections of the QC sample were performed at the onset of the run. The conditioning results are presented in Table 1 and show that the RSD of retention times and peak areas over the last 10 conditioning injections was <0.2% and <5%, respectively, for all compounds monitored except diclofenac. The larger variation of diclofenac may be a result of ionization effects due to co-eluting compounds as it eluted in a very crowded chromatographic region. Additionally, the retention times for all compounds stabilized quickly (first 20 injections in Figure 7, panels C and D). As such, despite the added complexity of the instrumental configuration, the instrument showed highly acceptable performance without increased need for conditioning.

#### 2.6.4. Retention Time and Peak Area Stability throughout All Injections

After conditioning a further 120+ injections of protein-precipitated plasma samples were performed to monitor the stability of the system over a simulated analytical run with ca 100 samples. Retention time and peak area stabilities for the full run are illustrated in Figure 7. In the HILIC separation, the retention times are highly stable with a moderate drift in peak area intensities over the entire run. In total, all compounds separated using HILIC exhibited an RSD ≤0.23% and ≤11% in retention times and peak areas, respectively. For the RPLC separation of the trapped compounds a continuous, but small, drift in retention times was observed across the full run with a similar downward trend in peak areas as that observed for the HILIC separated compounds. The peak area of diclofenac is the most varying of all monitored species (as was seen also in the conditioning step) which appears to increase towards the end of the run. In total, the RSD for the compounds collected in the heart-cut and separated using RPLC was ≤0.88% and ≤26.3% (≤5.66% excluding diclofenac) in retention times and peak areas, respectively. Again, it was observed that the chromatographic region where diclofenac eluted was very crowded with other peaks (data not shown) which may be a plausible cause of this increased variability in diclofenac specifically. For this reason, the gradient used in the application to real samples was modified to increase peak distribution. Based on the results obtained, from the perspective of repeatability in retention times and peak response, we can conclude that the drift in performance observed is not greater than what can be regarded as acceptable if the samples are analyzed using single-column untargeted methods [27,32] and can therefore be regarded as fit for purpose.

#### 2.6.5. Carry Over

Another important factor to consider in any method, but even more so in this case, is carry over. Considering the complexity of the instrumental configuration along with many reversals of flow directions, this need to be evaluated to ensure all channels are properly flushed between injections. The carry over of the test compounds is given in Table 2 as the percent peak area of the peak detected at the same retention time in the blank injection to the peak area in the last QC injection. The results of this test show that the carry over of all the test compounds used is at or lower than 3.31% of that of the last QC injection.

#### 2.6.6. Linearity

**Figure 8:**
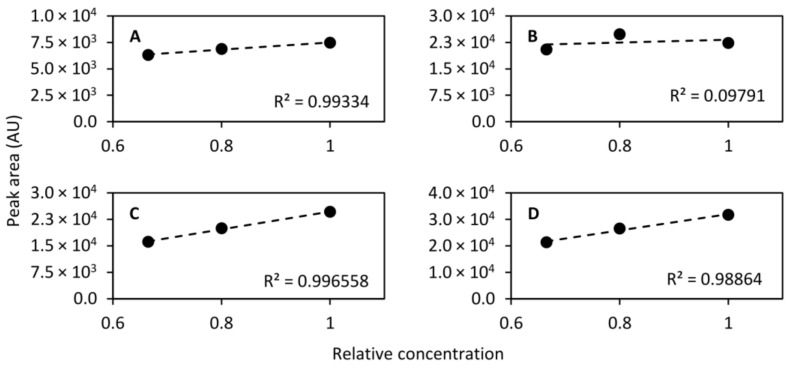
Linearity of acetylcholine (*m/z* 146.117, (**A**)), acetylcarnitine (*m/z* 204.123, (**B**)), serotonin (*m/z* 160.076, (**C**)), and phenylalanine (*m/z* 120.081, (**D**)) determined using the QC sample undiluted and diluted to 2/3 concentration and 4/5 concentration. Linear models were fitted to the mean peak areas of 17, 17, and 35 injections of the 4/5, 2/3 and 1 concentration samples, respectively.

**Figure 9:**
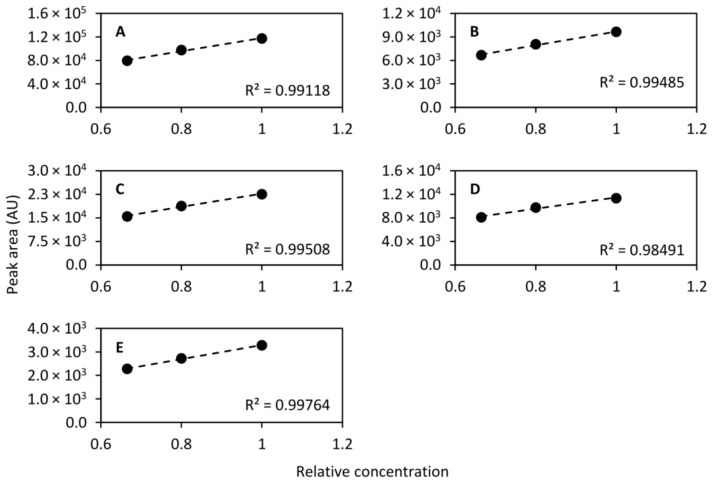
Linearity of caffeine (*m/z* 195.088, (**A**)), theobromine (*m/z* 181.072, (**B**)), theophylline (*m/z* 181.072, (**C**)), 2-aminobenzoic acid (*m/z* 120.045, (**D**)), and diclofenac (*m/z* 214.042, (**E**)) determined using the QC sample undiluted and diluted to 2/3 concentration and 4/5 concentration. Linear models were fitted to the mean peak areas of 17, 17, and 35 injections of the 4/5, 2/3 and 1 concentration samples, respectively.

While the dynamic range differs dramatically between different chemical species, it should be linear within a short span. To that end, diluted QC samples were prepared by diluting to 2/3 (1:2 dilution, *v/v*) and 4/5 (1:4 dilution, *v/v*) concentration and were injected at even intervals along with the QC sample. In total, the diluted QC samples were injected 17 times throughout the analytical run and the normal QC sample was injected 45 times, including conditioning injections. For the calculation of linearity only the last 10 conditioning injections were included giving a total of 35 injections. The linearity was then calculated using the mean peak area of each of the standard compounds monitored. Figure 8 shows the linearity of the compounds that were separated on the HILIC column and Figure 9 shows the linearity of the compounds collected in the heart-cut and separated on the RPLC column. As can be seen in the figures, eight out of nine compounds exhibited excellent linearity within the tested concentration range.

#### 2.6.7. Back Pressure Stability

**Figure 10:**
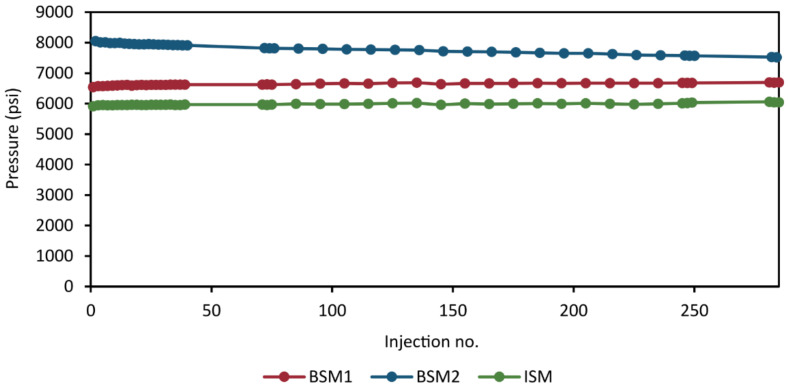
Instantaneous back pressure at the time point of maximum back pressure of the HILIC separation (BSM1), the RPLC separation (BSM2), and the loading of the sample loop to the trap column (ISM). For comparative purposes, for each pump, the plotted value is sampled from the same time point.

With the addition of many, narrow channels in the valves and the capillaries, the risk of build up of sample components and salts increases. For this reason, another important aspect to consider is how the system back pressure evolve during the analytical run. A slight increase in back pressure can be expected as more and more samples are injected. However, the increase must be marginal to avoid risk of deteriorating chromatographic performance. Sudden changes or a significant drift in back pressure may indicate a problem with the method. We monitored the back pressure during the validation analysis to ensure that it remained stable throughout. In Figure 10, the instantaneous maximum back pressures for the HILIC separation (BSM1), RPLC separation (BSM2), and the loading and dilution of the heart-cut to the trap column (ISM) are plotted as a function of injection number for each pump.

The back pressure of all pumps remained within reasonable stability throughout the analytical run with a marginal increase of ca 100 psi (~1.6%) in the BSM1 and ISM pumps, no increase was observed in BSM2.

#### 2.6.8. Summary of the Validation

As has been presented the system remains stable throughout 143 injections of protein-precipitated plasma. The repeatability across the entire analytical run in terms of both retention time and peak areas is high. We have also shown that the back pressure remains stable, indicating that the additional connections and narrow channels in the valves do not lead to accumulation of sample material components. Thus, we can conclude that the system is fit for purpose for use in untargeted metabolomics analysis of protein-precipitated blood plasma and should therefore perform equally well or better for perilymph samples.

### 2.7. Analysis of Guinea Pig Perilymph Samples in an Untargeted Metabolomics Study

#### 2.7.1. Modifications to the HILIC and RPLC Separation Gradients

**Figure 11:**
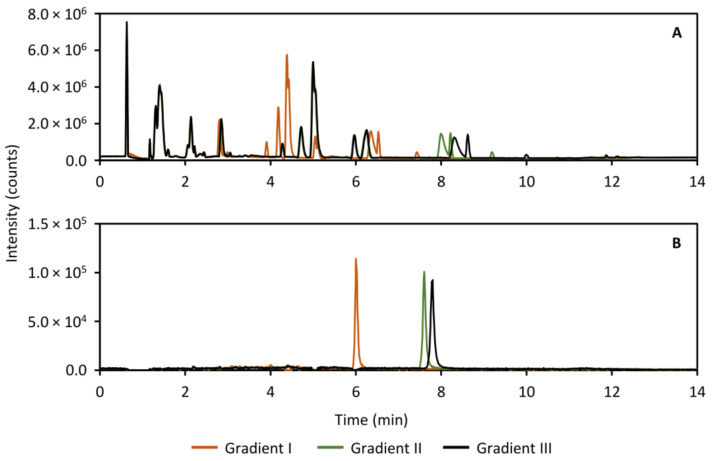
Full scan base peak ion trace (*m/z* 30–800, panel (**A**)) and extracted ion chromatogram of acetylcholine (*m/z* 146.117, panel (**B**)) showing three tested HILIC separation gradients for analyzing the guinea pig perilymph samples with the purpose of increasing peak distribution. The final gradient used in the analysis of the perilymph samples was gradient III (black traces in (**A**,**B**)) as this showed an overall improvement to retention.

**Figure 12:**
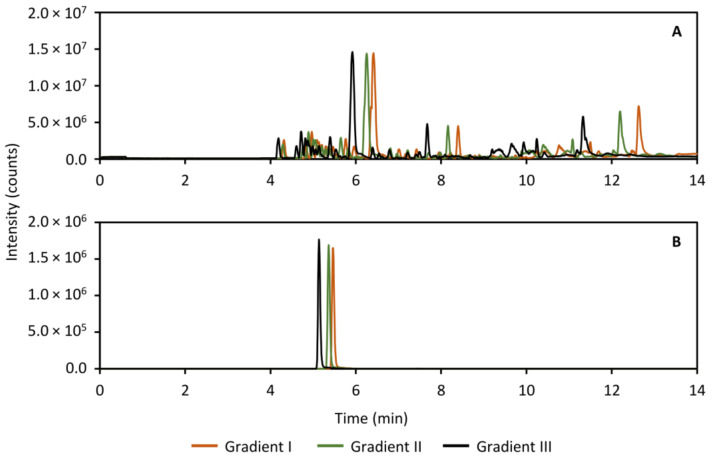
Full scan base peak ion trace (*m/z* 30–800, panel (**A**)) and extracted ion chromatogram of ketamine (*m/z* 238.0993, panel (**B**)) showing three tested RPLC separation gradients for analyzing the guinea pig perilymph samples with the purpose of increasing peak distribution. The final gradient used in the analysis of the perilymph samples was gradient III (black traces in (**A**,**B**)) as this showed an overall improvement.

During the validation experiments, it became obvious that the peak distribution with the gradient that was optimized for the standard compounds, needed improvement. For this reason, the gradient conditions for both the HILIC separation as well as the analysis of the heart-cut was modified with the aim of further distributing the peaks along the time axis. In the HILIC separation, the peaks did not exhibit sufficient retention in the gradient to fill out the chromatogram, and so the goal was to increase overall retention. The results are presented in Figure 11, which shows the base peak ion (BPI) trace (Figure 11A) of a separation of perilymph as well as the extracted ion chromatogram (XIC) of acetylcholine as an example (Figure 11B). In contrast, in the RPLC separation of the heart-cut, the retention was overall too high, leading to the peaks collecting at the end of the chromatogram. To distribute the peaks more, the goal here was therefore to reduce overall retention. The results of the RPLC gradient screen is shown in Figure 12, showing the BPI trace (Figure 12A) and the XIC of ketamine, the anesthetic in the study as an example (Figure 12B). In both cases, gradient III (black traces in Figure 11 and Figure 12) was used in the analysis of the perilymph samples.

#### 2.7.2. Univariate Quality Control

The univariate quality control of the analysis of the guinea pig perilymph samples, presented in Table 3, was performed in much the same way as in the validation experiments described previously. A selection of metabolite ions spanning the full chromatogram were monitored. Unfortunately, no peaks that were not present in the blank sample injection could be detected in negative mode in the collected heart-cut. Thus, no QC data are available for this mode. In HILIC with positive ionization, a large number of known metabolites were detected and used to monitor the system during the analytical run. All compounds exhibited retention time and peak area RSD values less than or equal to 0.22% and 9.78%, respectively. In HILIC with negative ionization, the retention time and peak area RSD values were less than or equal to 0.20% and 11.1%, respectively. In the RPLC separation of the collected heart-cut with positive ionization, a number of compounds are detected and used to evaluate the variability across the analytical run. Ketamine and its main metabolite norketamine is detected, which is the anesthetic used in the study. The animals were also given bupivacaine and xylazine which are also detected in the RPLC separation. In addition to these a number of putatively identified compounds as well as compounds that could not be identified were used. Over the full analytical run, the RSD of retention times and peak areas in the QC sample injections ranged from 0.10% to 0.28% and 3.47% to 28.6%, respectively. Even though no data are available for RPLC separation in the negative mode, we believe that the data collected from the other modes can attest to the stability of the system during the analytical run. Furthermore, the compounds detected in the RPLC separation are spread over most of the chromatogram, attesting to the wide range of compounds that can be detected in addition to the HILIC separation.

#### 2.7.3. Multivariate Quality Control

**Figure 13:**
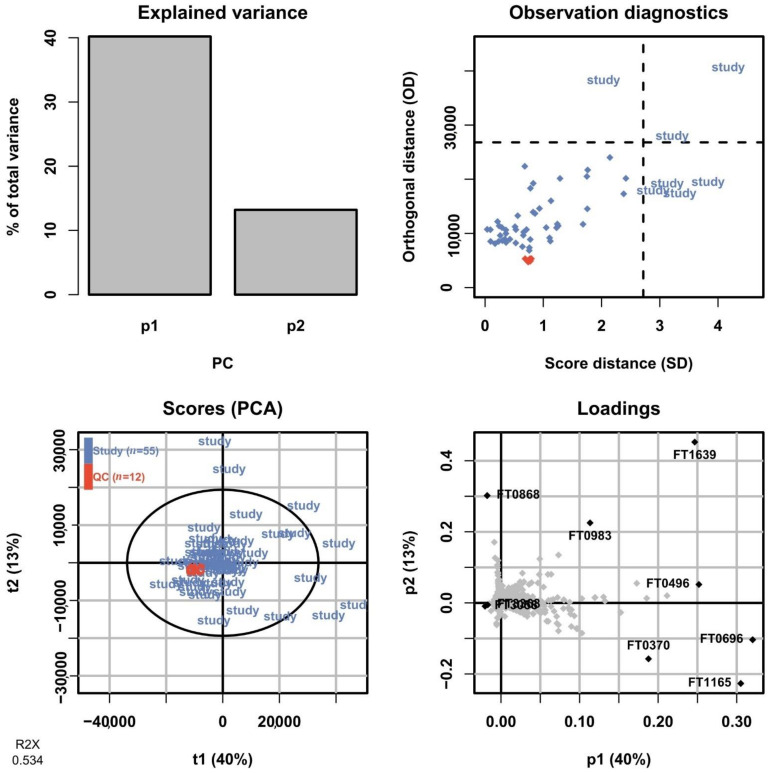
Summary of the PCA model fitted on HILIC-positive data of all sample groups including QC sample injections. In the scores plot (lower left), the QC sample injections (orange) are clustered tightly at the center of the plot, indicating low technical variation in relation to the biological variation.

**Figure 14:**
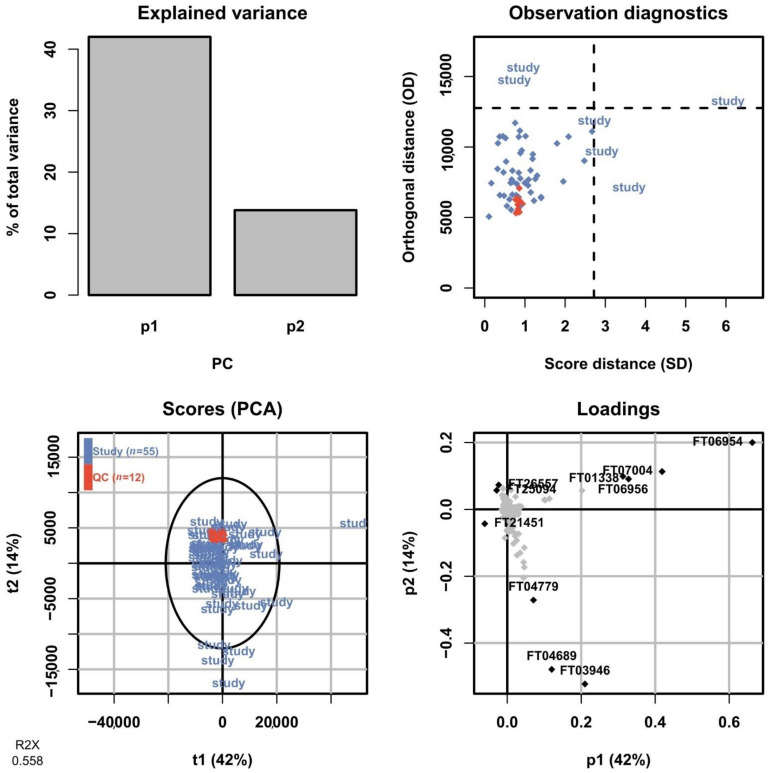
Summary of the PCA model fitted on the RPLC-positive data of all sample groups including QC sample injections. In the scores plot (lower left), the QC sample injections (orange) are clustered tightly at the center of the plot, indicating low technical variation in relation to the biological variation.

In untargeted metabolomics, a commonly employed method to assess the overall variation in the dataset is by fitting a PCA model to the full dataset including the QC sample injections. The PCA scores plot allows the technical variation exhibited by the QC sample injections to be assessed relative to the biological variation between study samples. As the QC sample is prepared from pooling aliquots from all samples, it should show up somewhere in the center of the study samples and preferably show a low spread in relation to them. After processing the data and removing features only present in the blanks and with an intensity RSD <30% across QC sample injections, a total of 3266 and 894 features were retained in the positive mode for HILIC and RPLC, respectively, and used to fit PCA models. In Figure 13, a summary of the PCA model for the HILIC-positive ionization is shown for the first two components corresponding to 40% and 13% of the total variation in the dataset, respectively. The scores plot shows the QC sample injections clustered very tightly close to the center of the plot, indicating a low technical variation in the HILIC analysis. The corresponding PCA model summary for the RPLC separation of the collected heart-cut is presented in Figure 14. Here, the total variation described by the model in the first and second components is similar at 42% and 14%, respectively. The QC sample injections are, again, clustered tightly in the center of the plot, indicating that the technical variation is small in relation to the biological variation here as well. The results of the full untargeted metabolomics analysis will be presented elsewhere.

## 3. Materials and Methods

### 3.1. Chemicals

All water used was purified using a Milli-Q™ water system from MilliPore (Bedford, MA, USA). Formic acid (LC–MS grade) and ammonium formate (LC–MS grade) was obtained from Fisher Scientific (Zurich, Switzerland). Acetonitrile (LC–MS grade) was purchased from Fisher Scientific. Acetonitrile used for protein precipitation of biological samples was kept on ice throughout the sample preparation procedure. Analytical standards of acetylcholine and serotonin were purchased from VWR International AB (Stockholm, Sweden) and 2-aminobenzoic acid, acetylcarnitine, caffeine, diclofenac, phenylalanine, theophylline, theobromine were purchased from Sigma-Aldrich (Steinheim, Germany). Pooled human blood plasma (Li-Hep) from anonymous healthy donors for the validation experiments was purchased from 3H Biomedical AB (Uppsala, Sweden).

### 3.2. Instrumentals

Chromatography was performed using a Waters ACQUITY I-class UPLC system (Waters Corp., Milford, MA, USA) composed of two binary pumps (ACQUITY BSM, Waters Corp., Milford, MA, USA), an isocratic pump (A CQUITY ISM, Waters Corp., Milford, MA, USA), a flow-through-needle sample manager (ACQUITY SM-FTN, Waters Corp., Milford, MA, USA), a column manager with customized configuration (ACQUITY CM, Waters Corp., Milford, MA, USA), three 6-port VICI valves (VICI C72X-6676EH, Waters Corp., Milford, MA, USA), a 50 µL filter mixer unit (Waters Corp., Milford, MA, USA), and a 200 µL Rheodyne sample loop (IDEX Health & Science, LLC, Rhonert Park, CA, USA). Detection was performed using a Synapt G2-S QTOF HRMS instrument (Waters Corp., Milford, MA, USA). Chromatographic separation of the hydrophilic compounds was performed on a Waters ACQUITY BEH Amide (100 × 2.1 mm i.d., 1.7 µm particle size, Waters Corp., Milford, MA, USA) column fitted with a Waters VanGuard BEH Amide (5 × 2.1 mm i.d., 1.7 µm particle size, Waters Corp., Milford, MA, USA) pre-column. Chromatographic separation of the lipophilic fraction collected in the analysis of hydrophilic compounds was achieved on a Waters ACQUITY HSS T3 (100 × 2.1 mm i.d., 1.8 µm particle size, Waters Corp., Milford, MA, USA) column fitted with a Waters ACQUITY VanGuard HSS T3 (5 × 2.1 mm i.d., 1.8 µm particle size, Waters Corp., Milford, MA, USA) pre-column. The lipophilic compounds collected in the HILIC separation were trapped on a Waters Oasis HLB trap column (20 × 2.1 mm i.d., 5 µm particle size, Waters Corp., Milford, MA, USA).

### 3.3. Software

The LC–MS equipment was controlled using MassLynx (v4.1, Waters Corp., Milford, MA, USA) configured with the Waters Pump Control (Waters Corp., Milford, MA, USA) LC-pump drivers loaded to facilitate control of multiple pumps. The TargetLynx (v4.1, Waters Corp., Milford, MA, USA) module for MassLynx was used to determine peak areas and retention times in the targeted analysis. Any further data analysis was performed either in Excel 2016 (Microsoft, Redmond, WA, USA) or in the R statistical language environment (v3.6.1). Conversion of Waters proprietary data format into NetCDF was performed using Databridge (v3.5, Micromass UK Ltd., Manchester, UK). Peak picking, retention time alignment, and correspondence were performed using the R-package XCMS (v3.12.0) [33]. Multivariate analysis was performed using the R-package ropls (v1.22.0). Batch and drift correction was performed using the batchCorr R-package (v0.2.5) [34].

### 3.4. Validation of the Developed Method for Use in Untargeted Metabolomics

#### 3.4.1. Experimental Design

A set of plasma samples were prepared from human blood plasma and analyzed in an analytical run of 140+ injections. The plasma samples were spiked with the same compounds used in the standard mix during method development and are presented in Table 4. In short, a QC sample was prepared by pooling multiple aliquots of protein-precipitated blood plasma. Aliquots from the QC sample were then further diluted to 4/5 (QCd1) and 2/3 (QCd2) concentration with 95:5 acetonitrile:water (*v/v*).

#### 3.4.2. Protein Precipitation of Human Blood Plasma

All sample preparation was performed on ice. To 250 µL thawed blood plasma, 15 µL standard mixture was added and vortexed 5 s. The standard mixture contained 2-aminobenzoic acid (71.0 µM), acetylcarnitine (0.347 µM), acetylcholine (3.95 µM), caffeine (3.42 µM), diclofenac (5.44 µM), phenylalanine (88.0 µM), serotonin (8.91 µM), theobromine (9.32 µM), and theophylline (9.04 µM) prepared from stock solutions and diluted with water. Proteins were precipitated by addition of 750 µL cold acetonitrile and the samples were then vortexed for 10 s, placed in the refrigerator (ca 4 °C) for 30 min and then centrifuged (21,000× *g*, 4 °C, 15 min). The supernatant was carefully collected and evaporated at reduced pressure for 3.5 h until dry. The residue was then dissolved in 200 µL 95:5 acetonitrile:water (*v/v*).

### 3.5. Sequential LC–MS Analysis of Hydrophilic and Lipophilic Component of Biological Samples

The mobile phases used for the HILIC separation was (A) 95:5 acetonitrile:water (*v/v*) with 5 mM ammonium formate and 0.065 vol% formic acid and (B) 5:95 acetonitrile:water (*v/v*) with 5 mM ammonium formate and 0.065 vol% formic acid. The mobile phases used for the RPLC separation was (A) 100% water with 0.1 vol% formic acid and (B) 100% acetonitrile with 0.1 vol% formic acid. The column temperatures were thermostated at 45 °C and 25 °C for the HILIC and RPLC column, respectively, while the trap column was kept at ambient temperature. The solvent used to dilute the collected heart-cut was 100% water with 0.1 vol% formic acid. The mobile-phase composition and flow rate of the three pumps throughout the method is given in Table 5.

**Figure 15:**
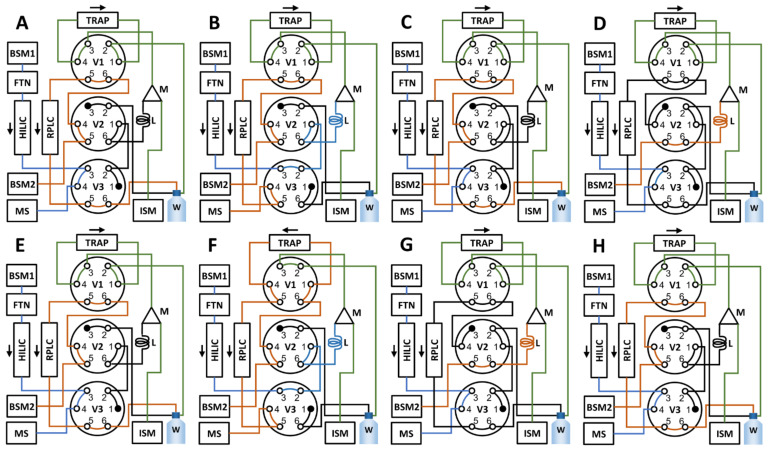
Schematics of the valve settings during the various steps of the sequential method. In summary, at 0.00, the sample is injected onto HILIC column (**A**); at 0.65 min, valve 3 is switched and the heart-cut collected (**B**); at 1.15 min, valve 3 is switched back (**C**); at 1.50 min, valve 2 is switched (**D**) and the collected heart-cut is loaded to the sample loop (marked L in the figure); at 10.00 min, valve 2 is switched back (**E**), the HILIC separation is then allowed to finish. At 15.00, valves 1 and 2 are switched (**F**), which starts the back-flushing of the trap to the RPLC column and this state is kept until 31.00, when all valves (1–3) are switched (**G**) to allow the mixer and trap channels to be flushed. Finally, at 34.00 min, valve 2 is switched (**H**), and the methods ends at 34.50 min.

A schematic summary of the valve settings throughout the different steps of the method is presented in Figure 15. At time 0.00 min, the sample is injected onto the HILIC column with setting A in Figure 15. After 0.65 min, valve 3 is switched (setting B, Figure 15), whereby the HILIC effluent is directed into the sample loop and collected. At 1.15 min, valve 3 is switched back (setting C, Figure 15), directing the HILIC effluent to the MS. At 1.50 min, valve 2 is switched (setting D, Figure 15), directing the BSM2 pump flow through the sample loop after which it mixes with the flow from the ISM pump allowing dilution of the collected volume before it is passed through the trap column. Valve 2 is switched back at 10.00 min (setting E, Figure 15) when the sample loop content has been displaced completely and this setting is kept for the duration of the HILIC separation. At 15.00 min, the HILIC separation is finished and valves 1 and 2 are switched (setting F, Figure 15) to allow the trap column to be back-flushed to the RPLC column using BSM2. During this stage, the HILIC column is flushed and re-equilibrated for the next injection using BSM1. After the RPLC separation is finished, at 31.00 min, all three valves are switched (setting G, Figure 15), whereby the sample loop, mixer, and trap column is flushed with 100% aqueous using BSM2 and ISM. At 34.00 min, valves 1 and 3 are switched (setting H, Figure 15) to ready the instrument for the next injection; and at 34.50 min, the method ends.

### 3.6. Untargeted Metabolomics Analysis of Guinea Pig Perilymph Samples

#### 3.6.1. Protein Precipitation of Perilymph Samples

To 1.0 µL of guinea pig perilymph, 9.0 µL water was added and the samples were centrifuged (21,000× *g*, 4 °C) for 5 min. Proteins were precipitated by addition of 40 µL cold acetonitrile and the samples were kept at 4 °C for 30 min. The samples were then centrifuged (21,000× *g*, 4 °C) for 15 min. A volume of 35 µL of the supernatant was removed and placed at −80 °C pending analysis. The QC sample was prepared by pooling 12 µL from each sample supernatant and placed at −80 °C pending analysis. Prior to analysis, the samples were thawed batch wise at 4 °C over night and centrifuged (21,000× *g*, 4 °C) for 5 min.

#### 3.6.2. Modified Gradients Used When Analyzing the Guinea Pig Perilymph Samples

The mobile phases used in the analysis of the perilymph samples were the same as that described in Section 3.5. The final gradient used in the HILIC separation of the guinea pig perilymph samples was 0.00–1.00 min 95%A (isocratic), 1.00–5.00 min 95%A–90%A (linear gradient), 5.00–10.00 min 90%A–80%A (linear gradient), 10.00–12.10 min 80%A–40%A (linear gradient), 12.10–14.00 min 40%A (isocratic), 14.00–14.10 min 40%A–95%A (linear gradient), and 14.10–14.50 min 95%A (isocratic). The final gradient used in the RPLC separation of the collected heart-cut from the perilymph samples was 15.00–16.00 min 100%A (isocratic), 16.00–23.50 min 100%A–2%A (linear gradient), 23.50–28.00 min 2%A (isocratic), 28.00–28.50 min 2%A–100%A (linear gradient), and 28.50–32.40 min 100%A (isocratic).

### 3.7. MS Detection

Calibration was performed using a solution of sodium formate according to manufacturer guidelines. For the validation experiments, centroided mass data were collected in the ESI+ mode with resolution set to resolution mode. For the analysis of guinea pig perilymph, centroided mass data were collected in both the ESI+ and ESI− modes with resolution set to resolution mode. Internal mass calibration was performed automatically using leucine-enkephalin (0.2 mM in 1:1 acetonitrile:water (*v/v*) + 0.1 vol% formic acid) infused at 10 µL/min and sampled every 60 s with a scan time of 0.5 s. Data were acquired in TOF MS mode, with a scan range of *m/z* 30–800, and a 0.5 s scan time. For a full list of MS tune settings, see Supplementary Materials Table S1.

### 3.8. Data Processing and Analysis

Following data conversion of the raw data into the netCDF format, XCMS was used for peak picking, retention alignment, and correspondence. All features only detected reliably in the blank injections by XCMS were then excluded. Additionally, features with intensities ≥70% of the mean QC sample intensity were also excluded. The features were then normalized using batchCorr which also filters features with an RSD >30% across QC sample injections after normalization. Principal component analysis was then performed on study samples and QC sample injections to study the technical variation in relation to the biological variation. For all full description of all parameters used in data processing, see Supplementary Materials Tables S2 and S3.

## 4. Conclusions

In conclusion, a method for single-injection sequential analysis of both the hydrophilic and lipophilic components of biological samples has been developed using commercially available equipment. The presented method allows easy adjustment to the dilution ratio of the collected heart-cut as it is loaded onto the trap column by relative adjustment of the flow rates of the binary and isocratic pumps used. This allows the method to be tailored to the application in terms of both time and solvent usage. Furthermore, we have shown that the method is fit for purpose for application in untargeted metabolomics analysis of biological samples. Peak area and retention time RSD values across over 140 injections of protein-precipitated plasma were ≤26.4% (≤11.0% if excluding diclofenac) and ≤0.88%, respectively, for both the HILIC and RPLC separations. These values are within the range commonly accepted in the field of untargeted metabolomics. The method was applied to the analysis of 55 samples of guinea pig perilymph in an untargeted metabolomics study of the attenuating effect of hydrogen gas on noise-induced hearing loss. We have shown that the method exhibits high performance over the full analytical run of real perilymph samples in terms of repeatability of both retention times and peak areas. The QC sample injections show a tight clustering, relative to the study samples, in the PCA scores plot, indicating a low technical variation in contrast with the biological variation. Overall, the most impactful parameters affecting the performance of the method were the gradients, flow rates and column temperatures used and we recommend that these parameters be carefully evaluated when developing methods such as the one presented in this paper.

## Figures and Tables

**Table 1:** Retention time- and peak area variation in the positive ESI mode for the last 10 conditioning injections for all compounds monitored.

Compound	Mode ^a^	Mean Retention Time (min)	Retention Time RSD (%)	Mean Peak Area (AU)	Peak Area RSD (%)
Acetylcholine	HILIC	1.59	0.00	8158.53	4.23
Acetylcarnitine	HILIC	4.71	0.07	20,584.15	3.88
Serotonin	HILIC	3.10	0.00	28,693.17	3.83
Phenylalanine	HILIC	4.39	0.07	34,879.93	3.10
Caffeine	RPLC	5.16	0.13	122,893.94	1.68
Theobromine	RPLC	4.53	0.11	10,203.70	3.81
Theophylline	RPLC	4.77	0.11	23,866.90	2.52
Diclofenac	RPLC	9.21	0.08	3958.71	24.4
2-aminobenzoic acid	RPLC	6.35	0.16	11,799.13	2.92

**^a^** HILIC indicates compounds that were separated on the HILIC column and RPLC indicates compounds that were collected, trapped, and subsequently analyzed using the RPLC method.

**Table 2:** Carry over measured as the relative peak area of any peak detected at the same retention time in the blank sample and given as % peak area of the last QC injection.

Standard Compound	Peak Area, Last QC Injection (AU)	Peak Area, Blank Injection (AU)	Carry Over
Acetylcholine	6837.1	n.d.	n.d.
Acetylcarnitine	21352.6	96.1	0.45%
Serotonin	20631.0	n.d.	n.d.
Phenylalanine	27144.9	n.d.	n.d.
Caffeine	108608.6	1214.5	1.1%
Theobromine	9178.5	303.8	3.3%
Theophylline	21561.0	245.0	1.1%
Diclofenac	2627.0	n.d.	n.d.
2-aminobenzoic acid	10742.1	n.d.	n.d.

n.d. = not detected in blank injection.

**Table 3:** Putatively identified metabolite ions used for quality control in the analysis of the guinea pig perilymph samples.

Compound	Chromatographic Mode	Detection Mode	Detected *m/z*	Molecular Formula^a^	rt (min)	Peak Area RSD (%)^b^	Retention Time RSD (%)^b^
Choline	HILIC	ESI+	104.1072	[C_5_H_14_NO]^+^	2.85	2.55	0.00
Nicotinic acid	HILIC	ESI+	124.0394	[C_6_H_5_NO_2_^+^H]^+^	3.66	4.06	0.16
Cytidine	HILIC	ESI+	266.0747	[C_9_H_13_N_3_O_5_^+^Na]^+^	3.97	2.20	0.13
Burytylcarnitine	HILIC	ESI+	232.1544	[C_11_H_21_NO_4_^+^H]^+^	4.37	2.04	0.07
Glycine betaine	HILIC	ESI+	118.0863	[C_5_H_12_NO_2_]^+^	5.04	2.04	0.09
Phenylalanine	HILIC	ESI+	120.0813	[C_8_H_10_N]^+^	5.14	1.73	0.06
Tryptophan	HILIC	ESI+	188.0713	[C_11_H_10_NO_2_]^+^	5.40	5.58	0.22
Carnitine	HILIC	ESI+	162.1129	[C_7_H_15_NO_3_^+^H]^+^	6.38	3.38	0.07
Taurine	HILIC	ESI+	126.0222	[C_2_H_8_NO_3_S^+^H]^+^	6.49	2.04	0.07
Creatine	HILIC	ESI+	132.0771	[C_4_H_9_N_3_O_2_^+^H]^+^	8.64	3.22	0.08
Glutamine	HILIC	ESI+	130.0496	[C_5_H_8_NO_3_]^+^	10.0	1.51	0.03
Arginine	HILIC	ESI+	175.1197	[C_6_H_14_N_4_O_2_^+^H]^+^	11.9	2.50	0.00
Uridine	HILIC	ESI−	243.0623	[C_9_H_12_N_2_O_6_^−^H]^−^	2.13	9.38	0.14
Taurine	HILIC	ESI−	124.0074	[C_2_H_7_NO_3_S^−^H]^−^	6.51	11.1	0.12
Glutamine	HILIC	ESI−	145.0619	[C_5_H_10_N_2_O_3_^−^H]^−^	10.0	9.78	0.04
Xanthine	HILIC	ESI−	151.0261	[C_5_H_4_N_4_O_2_^−^H]^−^	2.56	7.56	0.20
5’-Methylthioadenosine	RPLC	ESI+	298.0971	[C_11_H_15_N_5_O_3_S^+^H]^+^	4.51	21.8	0.25
Norketamine	RPLC	ESI+	224.0840	[C_12_H_14_NOCl^+^H]^+^	5.07	4.76	0.18
Ketamine	RPLC	ESI+	238.0993	[C_13_H_16_NOCl^+^H]^+^	5.14	4.76	0.18
U5.28	RPLC	ESI+	287.0786	[C_15_H_12_NO_5_^+^H]^+^	5.28	5.42	0.27
Phenylalanylcysteine	RPLC	ESI+	269.0984	[C_12_H_16_N_2_O_3_S^+^H]^+^	5.35	10.4	0.28
Xylazine	RPLC	ESI+	221.1115	[C_12_H_16_N_2_S^+^H]^+^	5.41	4.34	0.23
U5.45	RPLC	ESI+	251.1765	[C_14_H_22_N_2_O_2_^+^H]^+^	5.45	8.31	0.21
U5.75	RPLC	ESI+	240.1498	[C_15_H_17_N_3_^+^H]^+^	5.75	5.9	0.17
Bupivacaine	RPLC	ESI+	289.2287	[C_18_H_28_N_2_O^+^H]^+^	5.88	3.63	0.21
U6.73	RPLC	ESI+	267.1230	[C_15_H_22_S_2_^+^H]^+^	6.73	4.9	0.16
U7.34	RPLC	ESI+	288.2899	[C_17_H_37_NO_2_^+^H]^+^	7.34	3.47	0.18
U7.96	RPLC	ESI+	267.1234	[C_14_H_18_O_5_^+^H]+	7.96	28.6	0.14
Tetracosahexaenoic acid	RPLC	ESI+	357.2794	[C_24_H_36_O_2_^+^H]^+^	8.68	7.38	0.19
U8.68	RPLC	ESI+	478.3224	[C_30_H_41_N_2_O_3_^+^H]^+^	8.68	21.0	0.10

**^a^** Calculated using the Elemental Composition v. 4.0 tool of MassLynx 4.2, **^b^** RSD calculated over all QC sample injections (*n* = 12).

**Table 4:** Standard compounds that were used during the validation experiments and their concentration spiked into the blood plasma of the different types of samples utilized for validation. The QC sample was analyzed at intervals (*n* = 45, including 20 conditioning injections) during the entire analytical run, as is the practice in untargeted metabolomics. A set of diluted QC samples (QCd1 and QCd2 prepared as 1:2 and 1:4 dilutions, *v/v*, of the QC sample, respectively) were also analyzed at intervals (*n* = 17) throughout the analytical run.

Substance	Monoisotopic Mass (g/mol)	Concentration in the Spiked Samples (µM)		
		QC	QCd1	QCd2
2-aminobenzoic acid	137.0477	4.01	3.21	2.68
Acetylcarnitine	203.1158	0.0197	0.0157	0.0131
Acetylcholine	146.1181	0.224	0.179	0.149
Caffeine	194.0804	0.194	0.155	0.129
Diclofenac	295.0167	0.308	0.246	0.205
Phenylalanine	165.0790	4.98	3.98	3.32
Serotonin	176.0950	0.504	0.403	0.336
Theophylline	180.0647	0.511	0.409	0.341
Theobromine	180.0647	0.528	0.422	0.352

**Table 5:** Mobile-phase gradients in the heart-cut method during the validation experiments. BSM1 is used to elute the HILIC column, BSM2 is used to load the sample loop to the trap column and to elute the RPLC column, and ISM is used to dilute the collected heart-cut as it is displaced from the sample loop. The separation gradients for HILIC (BSM1, initial—14.50 min) and RPLC (BSM2, 15.00–33.90 min) is emphasized with bold text.

BSM1 Mobile-Phase Gradient			BSM2 Mobile-Phase Gradient			ISM Mobile-Phase Gradient	
Time (min)	Flow Rate (mL/min)	%A	Time (min)	Flow Rate (mL/min)	%A	Time (min)	Flow Rate (mL/min)
**Initial**	**0.400**	**95**	Initial	0.025	100	Initial	0.050
**1.00**	**0.400**	**95**	10.00	0.025	100	1.15	0.050
**11.50**	**0.400**	**65**	10.10	0.300	100	1.40	1.900
**12.10**	**0.400**	**40**	14.50	0.300	100	10.00	1.900
**14.00**	**0.400**	**40**	**15.00**	**0.300**	**100**	10.10	0.000
**14.10**	**0.400**	**95**	**16.50**	**0.300**	**100**	14.50	0.000
**14.50**	**0.400**	**95**	**25.00**	**0.300**	**2**	31.00	0.000
18.00	0.400	95	**28.00**	**0.300**	**2**	31.10	0.050
18.10	0.050	95	**28.50**	**0.300**	**100**	34.50	0.050
26.40	0.050	95	**33.90**	**0.300**	**100**		
26.50	0.400	95	34.00	0.025	100		
34.50	0.400	95	34.50	0.025	100		

## Data Availability

Data is available on request from the corresponding author with reservation for limitations due to ongoing research.

## Supplementary Materials

The following are available online at https://www.mdpi.com/article/10.3390/metabo11050295/s1, Figure S1: Peaks of caffeine, theobromine, theophylline, diclofenac, and 2-aminobenzoic acid as they elute off the trap column in backflush, Table S1: MS tune parameters used in the experiments, Table S2: XCMS parameters used when processing the perilymph data, Table S3: Parameters used for drift correction using the batchCorr R-package, Table S4: List of screened standard compounds with detected *m*/*z* and retention times.

## References

[1] Psychogios N., Hau D.D., Peng J., Guo A.C., Mandal R., Bouatra S., Sinelnikov I., Krishnamurthy R., Eisner R., Gautam B. (2011). The Human Serum Metabolome. PLoS ONE.

[2] Wishart D.S., Jewison T., Guo A.C., Wilson M., Knox C., Liu Y., Djoumbou Y., Mandal R., Aziat F., Dong E. (2012). HMDB 3.0—The Human Metabolome Database in 2013. Nucleic Acids Res..

[3] Gika H.G., Theodoridis G.A., Earll M., Wilson I.D. (2012). A QC Approach to the Determination of Day-to-Day Reproducibility and Robustness of LC–MS Methods for Global Metabolite Profiling in Metabonomics/Metabolomics. Bioanalysis.

[4] Ivanisevic J., Zhu Z.-J., Plate L., Tautenhahn R., Chen S., O’Brien P.J., Johnson C.H., Marletta M.A., Patti G.J., Siuzdak G. (2013). Toward Omic Scale Metabolite Profiling: A Dual Separation–Mass Spectrometry Approach for Coverage of Lipid and Central Carbon Metabolism. Anal. Chem..

[5] Vorkas P.A., Isaac G., Anwar M.A., Davies A.H., Want E.J., Nicholson J.K., Holmes E. (2015). Untargeted UPLC-MS Profiling Pipeline to Expand Tissue Metabolome Coverage: Application to Cardiovascular Disease. Anal. Chem..

[6] Gika H.G., Theodoridis G.A., Plumb R.S., Wilson I.D. (2014). Current Practice of Liquid Chromatography–Mass Spectrometry in Metabolomics and Metabonomics. J. Pharm Biomed. Anal..

[7] Want E.J., Wilson I.D., Gika H., Theodoridis G., Plumb R.S., Shockcor J., Holmes E., Nicholson J.K. (2010). Global Metabolic Profiling Procedures for Urine Using UPLC–MS. Nat. Protoc..

[8] Spagou K., Tsoukali H., Raikos N., Gika H., Wilson I.D., Theodoridis G. (2010). Hydrophilic Interaction Chromatography Coupled to MS for Metabonomic/Metabolomic Studies. J. Sep. Sci..

[9] Pirttilä K., Videhult Pierre P., Haglöf J., Engskog M., Hedeland M., Laurell G., Arvidsson T., Pettersson C. (2019). An LCMS-Based Untargeted Metabolomics Protocol for Cochlear Perilymph: Highlighting Metabolic Effects of Hydrogen Gas on the Inner Ear of Noise Exposed Guinea Pigs. Metabolomics.

[10] Fransson A.E., Kisiel M., Pirttilä K., Pettersson C., Videhult Pierre P., Laurell G.F.E. (2017). Hydrogen Inhalation Protects against Ototoxicity Induced by Intravenous Cisplatin in the Guinea Pig. Front. Cell. Neurosci..

[11] Videhult Pierre P., Pirttilä K., Pettersson C., Haglöf J., Kiesel M., Fransson A.E., Laurell G. Inhalation of Molecular Hydrogen Attenuates Acute Noise Trauma: A Preclinical in Vivo Study.

[12] Hara A., Salt A.N., Thalmann R. (1989). Perilymph Composition in Scala Tympani of the Cochlea: Influence of Cerebrospinal Fluid. Heart Res..

[13] Stanley S.M.R., Foo H.C. (2006). Screening for Basic Drugs in Equine Urine Using Direct-Injection Differential-Gradient LC–LC Coupled to Hybrid Tandem MS/MS. J. Chromatogr. B.

[14] Kwok W.H., Choi T.L.S., Tsoi Y.Y.K., Leung G.N.W., Wan T.S.M. (2017). Screening of over 100 Drugs in Horse Urine Using Automated On-Line Solid-Phase Extraction Coupled to Liquid Chromatography-High Resolution Mass Spectrometry for Doping Control. J. Chromatogr. A.

[15] Louw S., Pereira A.S., Lynen F., Hanna-Brown M., Sandra P. (2008). Serial Coupling of Reversed-Phase and Hydrophilic Interaction Liquid Chromatography to Broaden the Elution Window for the Analysis of Pharmaceutical Compounds. J. Chromatogr. A.

[16] Falasca S., Petruzziello F., Kretz R., Rainer G., Zhang X. (2012). Analysis of Multiple Quaternary Ammonium Compounds in the Brain Using Tandem Capillary Column Separation and High Resolution Mass Spectrometric Detection. J. Chromatogr. A.

[17] Greco G., Grosse S., Letzel T. (2013). Serial Coupling of Reversed-Phase and Zwitterionic Hydrophilic Interaction LC/MS for the Analysis of Polar and Nonpolar Phenols in Wine: Liquid Chromatography. J. Sep. Sci..

[18] Rajab M., Greco G., Heim C., Helmreich B., Letzel T. (2013). Serial Coupling of RP and Zwitterionic Hydrophilic Interaction LC-MS: Suspects Screening of Diclofenac Transformation Products by Oxidation with a Boron-Doped Diamond Electrode: Liquid Chromatography. J. Sep. Sci..

[19] Ortmayr K., Hann S., Koellensperger G. (2015). Complementing Reversed-Phase Selectivity with Porous Graphitized Carbon to Increase the Metabolome Coverage in an on-Line Two-Dimensional LC-MS Setup for Metabolomics. Analyst.

[20] Gargano A.F.G., Duffin M., Navarro P., Schoenmakers P.J. (2016). Reducing Dilution and Analysis Time in Online Comprehensive Two-Dimensional Liquid Chromatography by Active Modulation. Anal. Chem..

[21] Wang Y., Lehmann R., Lu X., Zhao X., Xu G. (2008). Novel, Fully Automatic Hydrophilic Interaction/Reversed-Phase Column-Switching High-Performance Liquid Chromatographic System for the Complementary Analysis of Polar and Apolar Compounds in Complex Samples. J. Chromatogr. A.

[22] Kittlaus S., Schimanke J., Kempe G., Speer K. (2013). Development and Validation of an Efficient Automated Method for the Analysis of 300 Pesticides in Foods Using Two-Dimensional Liquid Chromatography–Tandem Mass Spectrometry. J. Chromatogr. A.

[23] Cabooter D., Choikhet K., Lestremau F., Dittmann M., Desmet G. (2014). Towards a Generic Variable Column Length Method Development Strategy for Samples with a Large Variety in Polarity. J. Chromatogr. A.

[24] Loos G., Shoykhet K., Dittmann M., Cabooter D. (2017). Restriction Capillaries as an Innovative Mixing Unit for Intermediate Mobile Phase Exchange in Multidimensional Analysis. J. Chromatogr. A.

[25] Wang S., Zhou L., Wang Z., Shi X., Xu G. (2017). Simultaneous Metabolomics and Lipidomics Analysis Based on Novel Heart-Cutting Two-Dimensional Liquid Chromatography-Mass Spectrometry. Anal. Chim. Acta.

[26] Engskog M.K.R., Haglöf J., Arvidsson T., Pettersson C. (2016). LC–MS Based Global Metabolite Profiling: The Necessity of High Data Quality. Metabolomics.

[27] Broadhurst D., Goodacre R., Reinke S.N., Kuligowski J., Wilson I.D., Lewis M.R., Dunn W.B. (2018). Guidelines and Considerations for the Use of System Suitability and Quality Control Samples in Mass Spectrometry Assays Applied in Untargeted Clinical Metabolomic Studies. Metabolomics.

[28] Scheibe F., Haupt H. (1985). Biochemical Differences between Perilymph, Cerebrospinal Fluid and Blood Plasma in the Guinea Pig. Hear. Res..

[29] Thalmann I., Comegys T.H., Liu S.Z., Ito Z., Thalmann R. (1992). Protein Profiles of Perilymph and Endolymph of the Guinea Pig. Hear. Res..

[30] Leary Swan E.E., Peppi M., Chen Z., Green K.M., Evans J.E., McKenna M.J., Mescher M.J., Kujawa S.G., Sewell W.F. (2009). Proteomics Analysis of Perilymph and Cerebrospinal Fluid in Mouse: Perilymph Proteomics Analysis. Laryngoscope.

[31] Fujita T., Yamashita D., Irino Y., Kitamoto J., Fukuda Y., Inokuchi G., Hasegawa S., Otsuki N., Yoshida M., Nibu K. (2015). Metabolomic Profiling in Inner Ear Fluid by Gas Chromatography/Mass Spectrometry in Guinea Pig Cochlea. Neurosci. Lett..

[32] Dunn W.B., Wilson I.D., Nicholls A.W., Broadhurst D. (2012). The Importance of Experimental Design and QC Samples in Large-Scale and MS-Driven Untargeted Metabolomic Studies of Humans. Bioanalysis.

[33] Smith C.A., Want E.J., O’Maille G., Abagyan R., Siuzdak G. (2006). XCMS: Processing Mass Spectrometry Data for Metabolite Profiling Using Nonlinear Peak Alignment, Matching, and Identification. Anal. Chem..

[34] Brunius C., Shi L., Landberg R. (2016). Large-Scale Untargeted LC-MS Metabolomics Data Correction Using between-Batch Feature Alignment and Cluster-Based within-Batch Signal Intensity Drift Correction. Metabolomics.

