# Supplementary material for: Automated Sequential Analysis of Hydrophilic and Lipophilic Fractions of Biological Samples: Increasing Single-Injection Chemical Coverage in Untargeted Metabolomics
Source: Metabolites. 2021 May 5;11(5):295. doi: 10.3390/metabo11050295 (PMC8147996; doi:10.3390/metabo11050295)
Supplement: Supplementary file 1 [file metabolites-11-00295-s001.zip › metabolites-1170132-supplementary.pdf]

ELECTIONIC SUPPLEMENTARY MATERIAL

Automated sequential analysis of hydrophilic and lipophilic fractions of biological samples: Increasing single injection chemical coverage in untargeted metabolomics

Kristian Pirttilä [1,*], Göran Laurell [2], Curt Pettersson [1], Mikael Hedeland [1]

[1] Department of Medicinal Chemistry, Uppsala University, Uppsala, Sweden

[2] Department of Surgical Science, Uppsala University, SE-75123 Uppsala, Sweden

* Correspondence: kristian.pirttila@ilk.uu.se

Table S1. Tune settings used in the analysis of the human plasma samples and guinea pig perilymph samples.

**Parameter**	**ESI+**	**ESI-**
Caprillary voltage (kV)	1.5	1.9
Source temperature (°C)	120	100
Samling cone voltage (V)	30	40
Source offset voltage (V)	50	60
Source gas flow (mL/min)	0	0
Desolvation gas temperature (°C)	600	400
Cone gas flow (L/h)	25	30
Desolvation gas flow (L/h)	800	400
Nebulizer gas pressure (bar)	6	6

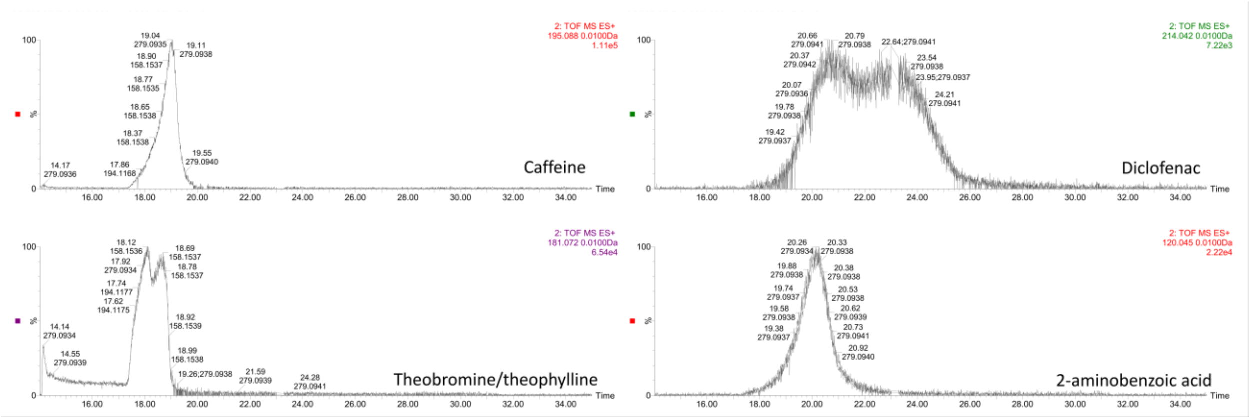

Figure S1. Peaks of caffeine, diclofenac, theobromine, theophylline, and 2-aminobenzoic acid as they elute off the trap column when backflushing the trap column without an RPLC column present. It is clear to see that the peaks, rather than eluting off the trap column as sharp peaks, are very poorly focused in the trap column, which leads us to believe that the majority of the peak focusing occur at the head of the secondary column, in this case the RPLC column.

Table S2. XCMS parameters used for peak detection, retention time alignment, correspondence, and peak filling

Processing step	Parameter	HILIC ESI+ data	RPLC ESI+ data
Peak detection (CentWave)	ppm	60	60
	peakwidth	{5, 50}	{5, 50}
	snthresh	10	10
	prefilter	{5, 1000}	{5, 1000}
	mzCenterFun	wMean	wMean
	integrate	2	2
	mzdiff	0.01	0.01
	fitgauss	TRUE	TRUE
	noise	200	200
	verboseColumns	TRUE	TRUE
Retention time correction (Obiwarp fitted using QC sample injections as subset)	binSize		
	response	1	1
	distFun	cor_opt	cor_opt
	gapInit	0.3	0.3
	gapExtend	2.4	2.4
	factorDiag	2	2
	factorGap	1	1
	localAlignment	FALSE	FALSE
	initPenalty	0	0
	subsetAdjust	average	average
Peak grouping (PeakDensity)	bw	2	2
	minFraction	0.8	0.8
	minSamples	1	1
	binSize	0.02	0.05
	maxFeatures	50	50
Missing peak filling (correspondence integration)	Default parameters used.		

Table S3. Parameters for the correctDrift() function in the batchCorr R-package. After drift correction, 3266 and 894 features from the HILIC and RPLC data sets, respectively, were kept after filtering features with CV ≥ 0.3 across QC sample injections.

Parameter	Set value for both HILIC and RPLC data
modelNames	VVE
G	seq(1,52,3)
CVlimit	0.3

Table S4. List of standard compounds screened during this work with the detected m/z in both positive and negative electrospray mode as well as retention times for both HILIC and RPLC separation. The HILIC column used was a Waters ACQUITY BEH Amide (100x2.1 mm i.d., 1.7 µm particle size) equipped with a Waters VanGuard BEH Amide (5x2.1 mm i.d., 1.7 µm particle size) pre-column and the RPLC column used was a Waters ACQUITY HSS T3 (100x2.1 mm i.d., 1.8 µm) with a Waters ACQUITY VanGuard HSS T3 (5x2.1 mm i.d., 1.8 µm particle size) pre-column.

Substance name	CAS	Monoisotopic mass [g/mol]	Detected m/z ESI+ T3	Detected m/z ESI- T3	Detected m/z ESI+ Amide	Detected m/z ESI- Amide	Amide rt (min)	T3 rt (min)
methylthioadenosyl, 5'-	2457-80-9	297.0895601	136.0627	134.0455	298.0983	134.0462	1.89	5.45
Acetylcarnitine	5080-50-2	203.115758	204.1243	N/D	204.1238	N/D	6.06	1.01
Acetylcholine	60-31-1	146.1181038	N/D	N/D	146.1176	N/D	2.08	N/D
Adenine	73-24-5	135.0544952	136.0626	134.0456	136.0622	134.046	2.515	0.71
Adenosine	58-61-7	267.0967539	136.0632	134.0456	136.0624	134.0463	3.275	0.87
ADP	20398-34-9	427.0294147	428.0359	426.0201	N/D	N/D	N/D	0.945
Alanine	56-41-7	89.04767847	N/D	N/D	N/D	N/D	N/D	N/D
Arginine	1119-34-2	174.1116757	175.1192	173.1024	175.1193	173.1029	9.735	0.63
Ascorbic acid	50-81-7	176.032088	N/D	N/D	N/D	N/D	N/D	N/D
Aspartic acid	56-84-8	133.0375077	N/D	132.0287	N/D	N/D	N/D	0.67
ATP	34369-07-8	506.9957452	N/D	N/D	N/D	N/D	N/D	N/D
Beta-Alanine	107-95-9	89.04767847	N/D	N/D	N/D	N/D	N/D	N/D
Betaine	107-43-7	117.0789786	118.0866	N/D	118.0869	N/D	5.72	0.75
Butyrylcarnitine	25576-40-3	231.1470582	232.1565	N/D	232.1576	N/D	4.8	4.35
Cadaverine	462-94-2	102.1156985	N/D	N/D	103.1235	N/D	8.96	N/D
Capric acid	334-48-6	172.1463299	N/D	171.1371	N/D	171.1377	0.78	15.98
Caprylic acid	124-07-3	144.1150298	N/D	143.106	N/D	143.1066	0.78	15.28
Carnitine	6645-46-1	161.1051934	162.1131	N/D	162.1137	N/D	6.64	0.67
Carnosine	305-84-0	226.1065903	227.1143	225.0979	227.1138	225.0977	10.005	0.6
Choline	67-48-1	104.1075391	104.1072	N/D	104.1073	N/D	3.76	0.65
Citric acid	77-92-9	192.0270026	N/D	191.0178	N/D	N/D	N/D	0.88
Citrulline	372-75-8	175.0956913	176.103	174.0867	198.0858	131.0811	8.44	0.67
CMP	63-37-6	323.051851	324.0591	322.0435	N/D	N/D	N/D	0.68

Substance name	CAS	Monoisotopic mass [g/mol]	Detected m/z ESI+ T3	Detected m/z ESI- T3	Detected m/z ESI+ Amide	Detected m/z ESI- Amide	Amide rt (min)	T3 rt (min)
Creatine	57-00-1	131.0694765	132.0774	130.0602	132.0775	130.0609	7.225	0.79
Creatinine	60-27-5	113.0589119	114.0666	112.0497	114.0667	112.05	3.225	0.76
CTP	36051-68-0	482.9845118	N/D	N/D	N/D	N/D	N/D	N/D
Cystine	56-89-3	240.0238483	241.0312	239.0148	N/D	N/D	N/D	0.66
Cytidine	65-46-3	243.0855205	112.0511	242.0765	266.0761	242.0767	5.26	0.7
Deoxyinosine	890-38-0	252.0858549	137.0465	251.0767	137.0463	251.0781	3.66	1.85
Deoxyribose	533-67-5	134.0579088	N/D	N/D	N/D	N/D	N/D	N/D
Dopamine	62-31-7	153.0789786	154.0862	152.07	N/D	152.0706	4.8	0.97
FAD	84366-81-4	785.1571345	786.1617	784.1489	786.1634	784.1498	10.3	3.01
Folic acid	59-30-3	441.1396814	295.0953	440.1319	295.0948	440.1319	7.28	6.8
Gamma aminobutyric acid	56-12-2	103.0633285	N/D	N/D	N/D	N/D	N/D	N/D
Glutamine	56-85-9	146.0691422	130.05	145.06	130.0496	145.0602	7.995	0.67
Glutathione	70-18-8	307.083806	308.0912	306.0751	N/D	N/D	N/D	0.71
Glutathione oxidized	27025-41-8	612.1519619	613.159	611.1439	N/D	N/D	N/D	0.87
Glycine	56-40-6	75.03202841	N/D	N/D	N/D	N/D	N/D	N/D
GMP	5550-12-9	363.057999	152.0572	362.0497	N/D	N/D	N/D	0.73
Histidine	5934-29-2	155.0694765	156.0768	154.0608	156.0766	154.0605	9.925	0.62
Homocysteine	454-29-5	135.0353992	136.044	134.0253	177.0695	N/D	5.69	0.745
Hydroxyproline	51-35-4	131.0582432	132.066	130.0494	132.0657	130.0495	7.19	0.705
Hypoxanthine	68-94-0	136.0385108	137.0465	135.0296	137.0461	135.0299	3.345	1.05
Inosine	58-63-9	268.0807695	137.0465	267.0724	137.0463	267.0725	4.64	1.62
Isobutyric acid	79-31-2	88.0524295	N/D	N/D	N/D	N/D	N/D	N/D
Isoleucine	73-32-5	131.0946287	132.1019	130.0854	176.0663	130.0857	5.875	1.87
Lactic acid	867-56-1	90.03169406	N/D	N/D	N/D	N/D	N/D	N/D
Leucine	61-90-5	131.0946287	132.102	130.0854	176.0663	130.086	5.005	1.87
Malic acid	97-67-6	134.0215233	N/D	133.0124	N/D	N/D	N/D	0.76
Malonic acid	141-82-2	104.0109586	N/D	N/D	N/D	103.0017	3.81	N/D

Substance name	CAS	Monoisotopic mass [g/mol]	Detected m/z ESI+ T3	Detected m/z ESI- T3	Detected m/z ESI+ Amide	Detected m/z ESI- Amide	Amide rt (min)	T3 rt (min)
Myoinositol	87-89-8	180.0633881	N/D	179.054	N/D	179.0546	8.04	0.66
NADH	606-68-8	665.1247717	666.1301	664.116	666.131	N/D	10.48	0.91
Niacinamide	98-92-0	122.0480128	123.0558	N/D	123.0559	N/D	1.22	0.86
Nicotinic acid	59-67-6	123.0320284	124.0398	122.0231	124.0398	122.0229	4.3	1.04
Palmitic acid	57-10-3	256.2402303	N/D	255.2194	N/D	255.2316	0.72	16.04
Palmitoylcarnitine	6865-14-1	399.3348589	400.3427	255.2318	400.3446	255.2321	3.015	16.04
Phenylalanine	63-91-2	165.0789786	120.0814	164.0698	120.0815	164.0708	5.56	2.82
Phosphocreatine	19333-65-4	211.035807	212.0429	N/D	212.0437	N/D	10.46	0.73
Phosphorylcholine	72556-74-2	184.0738695	184.074	N/D	184.0739	N/D	10.47	0.66
Proline	147-85-3	115.0633285	116.0707	114.0556	116.0704	114.0549	6.215	0.785
Putrescine	110-60-1	88.10004839	89.1073	N/D	89.1077	N/D	9.28	0.58
Pyridoxal	65-22-5	167.0582432	150.0557	166.0494	168.0658	166.0501	1.425	0.73
Pyruvic acid	127-17-3	88.01604399	N/D	87.0068	N/D	87.0069	3.98	1.06
Riboflavin	83-88-5	376.1382844	N/D	N/D	N/D	N/D	N/D	N/D
Ribose	50-69-1	150.0528234	N/D	N/D	N/D	N/D	N/D	N/D
Serine	56-45-1	105.0425931	N/D	104.0335	N/D	104.0339	8.06	0.66
Serotonin	153-98-0	176.094963	160.0768	N/D	160.0771	N/D	4.19	1.88
Spermidine	124-20-9	145.1578976	146.1652	N/D	N/D	N/D	N/D	0.56
Spermine	71-44-3	202.2157469	203.2236	N/D	N/D	N/D	N/D	0.54
Stearic acid	57-11-4	284.2715304	N/D	283.2621	N/D	N/D	N/D	14.32
Succinic acid	110-15-6	118.0266087	119.0353	117.0178	N/D	117.0179	4.33	0.98
Taurine	107-35-7	125.0146638	126.0221	124.0058	126.0219	124.0059	6.35	0.67
Thiamine	67-03-8	265.1123069	265.1124	N/D	144.0486	263.096	5.415	0.63
Thymine	65-71-4	126.0429274	127.0509	125.0339	127.0501	125.0342	1.56	1.57
Tryptophan	73-22-3	204.0898776	188.0714	203.081	188.0713	203.0814	5.59	4.86
Tyrosine	60-18-4	181.0738932	182.0815	180.065	182.0815	180.0653	6.415	0.88
Uric acid	69-93-2	168.02834	N/D	N/D	N/D	N/D	N/D	N/D
Uridine	58-96-8	244.0695361	267.0587	243.0612	113.0346	243.0611	3.38	0.87

Substance name	CAS	Monoisotopic mass [g/mol]	Detected m/z ESI+ T3	Detected m/z ESI- T3	Detected m/z ESI+ Amide	Detected m/z ESI- Amide	Amide rt (min)	T3 rt (min)
Valine	72-18-4	117.0789786	N/D	116.0696	N/D	N/D	N/D	0.75
Xanthine	69-89-6	152.0334254	N/D	N/D	N/D	N/D	N/D	N/D
Caffeine	58-08-2	194.080376	195.0881	N/D	195.0933	N/A	0.83	6.88
Ibuprofen	15687-27-1	206.13068	161.1323	205.1217	161.1318	N/A	0.77	16.16
hydroxybenzaldehyde, 4-	123-08-0	122.036779	123.0438	121.0284	123.043	N/A	0.83	6.975
acetyl-L-cysteine, N-	616-91-1	163.030314	N/D	162.0213	N/D	N/A	N/D	2.19
theobromine	83-67-0	180.064726	181.0729	N/D	181.0735	N/A	1.21	4.69
acetyl-D-tryptophan, N-	2280-01-5	246.100442	188.071	245.0923	188.0707	N/A	3.52	9.72
quinolinecarboxylic acid, 2-	93-10-7	173.047678	174.0548	172.0486	174.0549	N/A	3.22	6.035
Diclofenac	15307-86-5	295.016684	214.0424	250.0188	296.0238	N/A	0.95	16.05
aminobenzoic acid, 2-	118-92-3	137.047678	120.0447	136.0385	120.0445	N/A	0.98	7.38
theophylline	58-55-9	180.064726	181.0729	179.0573	181.0728	N/A	1.11	5.5
dihydroxybenzoic acid, 2,5-	490-79-9	154.026609	137.0232	153.0179	137.0342	N/A	1.05	5.655

N/D indicates a compound was not detected in the given mode.
